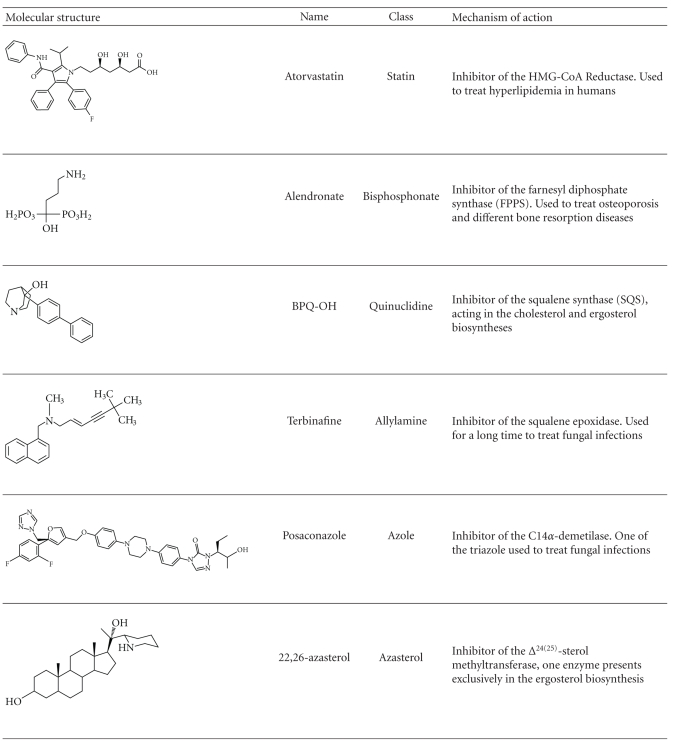# Sterol Biosynthesis Pathway as Target for Anti-trypanosomatid Drugs

**DOI:** 10.1155/2009/642502

**Published:** 2009-08-05

**Authors:** Wanderley de Souza, Juliany Cola Fernandes Rodrigues

**Affiliations:** ^1^Laboratório de Ultraestrutura Celular Hertha Meyer, Instituto de Biofísicia Carlos Chagas Filho, Universidade Federal do Rio de Janeiro, CCS-Bloco G, 21941-902, Rio de Janeiro, RJ, Brazil; ^2^Diretoria de Programas, Instituto Nacional de Metrologia, Normalização e Qualidade Industrial, Rio de Janeiro, RJ, Brazil

## Abstract

Sterols are constituents of the cellular membranes that are essential for their normal structure and function. In mammalian cells, cholesterol is the main sterol found in the various membranes. However, other sterols predominate in eukaryotic microorganisms such as fungi and protozoa. It is now well established that an important metabolic pathway in fungi and in members of the Trypanosomatidae family is one that produces a special class of sterols, including ergosterol, and other 24-methyl sterols, which are required for parasitic growth and viability, but are absent from mammalian host cells. Currently, there are several drugs that interfere with sterol biosynthesis (SB) that are in use to treat diseases such as high cholesterol in humans and fungal infections. In this review, we analyze the effects of drugs such as (a) statins, which act on the mevalonate pathway by inhibiting HMG-CoA reductase, (b) bisphosphonates, which interfere with the isoprenoid pathway in the step catalyzed by farnesyl diphosphate synthase, (c) zaragozic acids and quinuclidines, inhibitors of squalene synthase (SQS), which catalyzes the first committed step in sterol biosynthesis, (d) allylamines, inhibitors of squalene epoxidase, (e) azoles, which inhibit C14*α*-demethylase, and (f) azasterols, which inhibit Δ^24(25)^-sterol methyltransferase (SMT). Inhibition of this last step appears to have high selectivity for fungi and trypanosomatids, since this enzyme is not found in mammalian cells. We review here the IC50 values of these various inhibitors, their effects on the growth of trypanosomatids (both in axenic cultures and in cell cultures), and their effects on protozoan structural organization (as evaluted by light and electron microscopy) and lipid composition. The results show that the mitochondrial membrane as well as the membrane lining the protozoan cell body and flagellum are the main targets. Probably as a consequence of these primary effects, other important changes take place in the organization of the kinetoplast DNA network and on the protozoan cell cycle. In addition, apoptosis-like and autophagic processes induced by several of the inhibitors tested led to parasite death.

## 1. Introduction

Sterols are constituents of the cellular membranes that are essential for their normal structure and function. In mammalian cells, cholesterol is the main sterol found in the various membranes. However, other sterols predominate in eukaryotic microorganisms such as fungi and protozoa. In the case of trypanosomatids, for many years cholesterol was considered to be the major sterol. This was due to the fact that all biochemical analyses were undertaken in protozoa grown in complex media containing either brain, heart, or liver extracts and bovine serum. When the first biochemical analysis of lower trypanosomatids grown in chemically defined medium was carried out, it became clear that they synthesize ergosterol and not cholesterol [1]. Subsequently, it was shown that the trypanosomatids incorporate cholesterol from the culture medium or from the blood of infected animals through a typical endocytic process involving, in the case of epimastigotes of *Trypanosoma cruzi*, the formation of endocytic vesicles in the flagellar pocket and the cytostome [2, 3].

**Figure 1:**
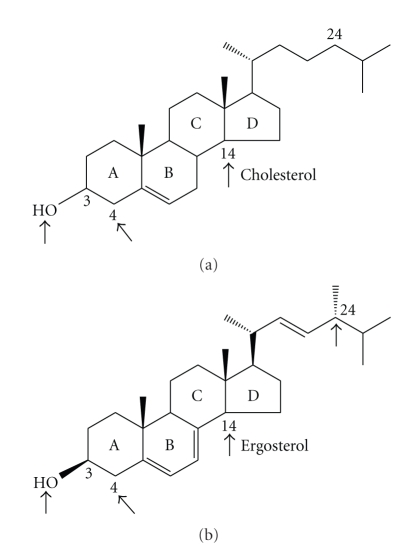
Molecular structures of cholesterol and ergosterol. The arrows indicate the parts of the molecules which have been shown to be essential for the growth of mammalian cells (cholesterol), fungi, and trypanosomatids (ergosterol and 24-methyl sterols).

It is now well established that an important metabolic pathway in fungi and in members of the Trypanosomatidae family is the sterol biosynthesis (SB) pathway. In these organisms, this pathway produces a special class of sterols, including ergosterol and other 24-methyl sterols, that is, required for their growth and viability, but is absent from mammalian host cells [4, 5]. Cholesterol and ergosterol differ in a few minor ways, as shown in Figure 1; cholesterol has only one double bond (Δ5(6)) in the B ring and has a fully saturated side chain without a methyl group at C24. It has been shown that some parts of the sterol molecule are important for its activity in cellular membranes. In the tetracyclic nucleus, the 3*β*–OH is obligatory for growth, whereas the presence of methyl groups at C14 or C4 do not allow growth. These two characteristics are essential for both cholesterol and ergosterol to support growth. However, certain characteristics of ergosterol that are absent in the cholesterol molecule, such as the presence of two double bonds in the B ring of the steroid nucleus, the presence of a *β* methyl at position 24, and the double bond at C22 in the side chain are essential for the growth of fungi and trypanosomatids.

The main sterols of the trypanosomatids comprise Δ^5,7^-compounds belonging to the C_28_-ergostane (24-methyl or methylene) or the C_29_-stigmastane (24-ethyl or ethylidine) groups [6–9]. In *Leishmania* amastigotes and promastigotes, the predominant sterol includes ergosta-5,7,24(24^1^)-trien-3*β*-ol (5-dehydroepisterol), although ergosta-118 7,24(24^1^)-dien-3*β*-ol (episterol) and ergosta-5,7,22-trien-3*β*-ol (ergosterol, the major fungal sterol) are present in smaller amounts. Stigmastane-related sterols comprise 5% of the total sterols in promastigotes; which can go as high as 20% in amastigotes of some species [9–12]. In contrast, *T. cruzi* epimastigotes contain around 40% ergosterol and ergosta-5,7-dien-3*β*-ol, and an appreciable amount, around 30%, of stigmasta-5,7-dien-3*β*-ol, and stigmasta-5,7,22-trien-3*β*-ol [8, 13]. In the case of amastigote forms, the sterol content is completely different since *T. cruzi* apparently does not produce Δ^5,7^-sterols, which are replaced by ergosta-7-en-3*β*-ol and 24-ethylidinecholest-7-en-3*β*-ol, indicating the absence of Δ^5^-desaturase activity [14]. The content of sterols in *Trypanosoma brucei* differs from other trypanosomatids, where the bloodstream form contains predominantly cholesterol incorporated from the medium through a receptor-mediated endocytic process, which apparently suppresses *de novo* synthesis of C_28_-sterols [15], even though it has mRNA coding for enzymes involved in ergosterol biosynthesis [16]. In the procyclic form, the sterol content is different from the bloodstream form, with the major component in the total membranes being ergosterol, with some amount of cholesterol [16]. 

**Figure 2:**
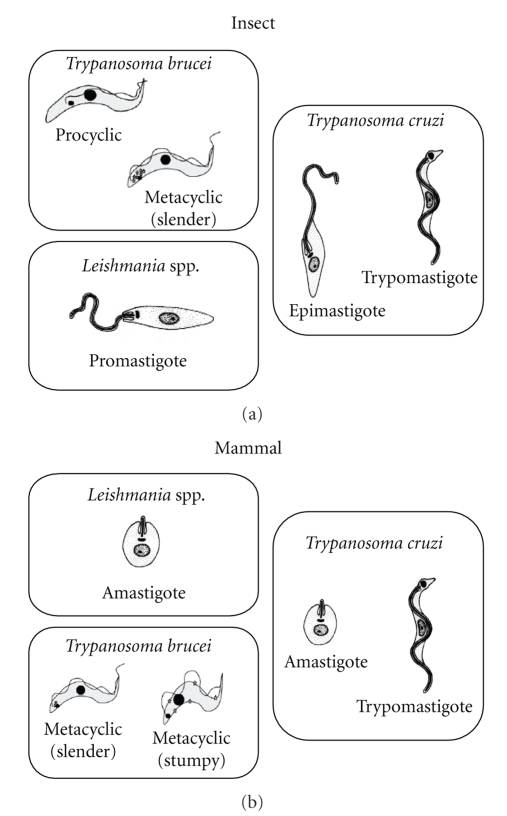
Schematic representation of main morphologies found during the life cycle of some members of the Trypanosomatidae family in the invertebrate host (insect) and vertebrate host (mammal).

In the context of this review describing the effect of sterol biosynthesis inhibitors in members of the Trypasonomatidae family, it is important to show a schematic representation of the main developmental stages found in the invertebrate host (insect) and vertebrate host (mammal) for *Trypanosoma brucei*, *Trypanosoma cruzi*, and *Leishmania spp* (Figure 2) [17–20]*.*

## 2. Comparison of the Sterol Biosynthesis Pathways in Mammals, Fungi, and Trypanosomatids

**Figure 3:**
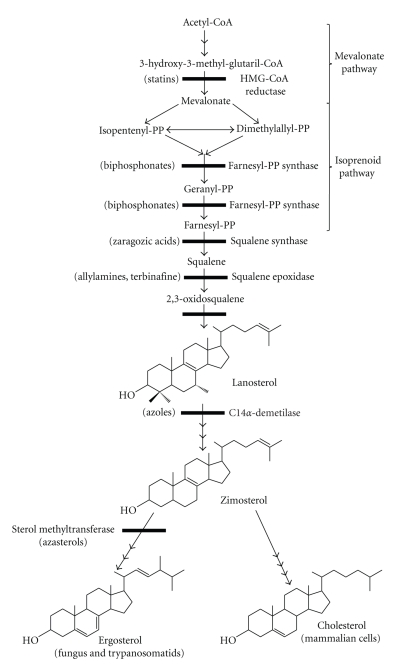
The biosynthesis of ergosterol and cholesterol showing the main steps, the enzymes involved, and the known inhibitors.

A comparative schematic diagram of the sterol biosynthesis pathway in eukaryotes is shown in Figure 3. At least 20 metabolic steps are necessary to synthesize such sterols as cholesterol and ergosterol, with some steps involving specific enzymes that differ between mammalian cells and microorganisms such as fungi and trypanosomatids. Some of these enzymes have been extensively studied, both as targets for the development of new drugs that interfere with parasite growth without severe effects on host cells and also as a means of reducing the high levels of endogenous cholesterol in mammalian cells. 

The carbon skeleton of the sterol molecule is derived from acetyl-CoA, with the exception to the presence of the C24 methyl group in the ergosterol side chain. The first reactions in the biosynthetic pathway involve condensation of two acetyl-CoA units to form acetoacetyl-CoA, followed by the addition of a third unit to form 3-hydroxy-3-methylglutaryl-CoA (HMG-CoA), which is then reduced by NADPH to give mevalonic acid. These three steps comprise the mevalonate pathway and are catalyzed by the cytosolic enzyme acetoacetyl-CoA thiolase, and two mitochondrial enzymes in yeasts and trypanosomatids, HMG-CoA synthase, and HMG-CoA reductase [21–23]. By contrast, the initial steps of cholesterol biosynthesis in mammalian cells occur mainly in the cytosol, with the later steps occurring in the endoplasmic reticulum (ER). On the other hand, several of the enzymes have been reported to exist in peroxisomes and in glycosomes [23–25].

After the mevalonate pathway, the next steps constitute the isoprenoid pathway. Isoprenoids are the most diverse and abundant compounds present in nature, and are essential components of all organisms due to a variety of roles in different biological processes. First, mevalonate is converted to isopentenyl diphosphate (IPP) by two phosphorylation reactions followed by one decarboxylation. Subsequently, isomerization of IPP by isopentenyl diphosphate isomerase produces dimethylallyl diphosphate (DMAPP). After that, longer isoprenoids are formed by a consecutive condensation of IPP with DMAPP and geranyl diphosphate (GPP) to produce the 15-carbon isoprenoid compound known as farnesyl diphosphate (FPP) in two reactions catalyzed by the enzyme farnesyl diphosphate synthase (FPPS). All these reactions together constitute the isoprenoid pathway, which is present in almost all organisms investigated so far [25]. FPP, which is the last product of isoprenoid biosynthesis, is the substrate for enzymes catalyzing the first committed step for the biosynthesis of sterols, ubiquinones, dolichols, heme A, and prenylated proteins. The enzymes involved in the isoprenoid pathway are located in different subcellular compartments such as the cytosol [26], mitochondria [27, 28], plastids in plant cells [26], and the peroxisome in animals [29]. For example, in *Leishmania major,* FPPS is located in the cytosol [30], while in *Toxoplasma gondii* it appears partially in the mitochondrion without any co-localization in rhoptries or apicoplast [31].

After the isoprenoid pathway, the next two reactions comprise the first committed step in sterol biosynthesis. These are catalyzed by the enzyme squalene synthase, which promotes a head-to-head condensation of two molecules of farnesyl diphosphate to produce squalene. In the first reaction, presqualene pyrophosphate (PPP) is produced by the loss of an inorganic pyrophosphate. This is converted to squalene in the second reaction in presence of NADPH, an essential cofactor required to drive this conversion [32]. In mammalian cells and in yeasts, squalene synthase is associated with the endoplasmic reticulum [33]. In *L. major*, its localization is still controversial. The presence of the signal sequences PTS1 and PTS2, typical of glycosomal proteins, indicates localization to the glycosome [34], while biochemical analysis of subcellular fractions of *T. cruzi* and *Leishmania spp.* showed that it could be found in the mitochondrion and microsomal fractions [35–37].

After production of squalene, sterol biosynthesis continues with the synthesis of 2,3-oxidosqualene (or squalene epoxide) in a reaction catalyzed by the enzyme squalene epoxidase. This is the first step in the conversion of the 30-carbon chain squalene to the tetracyclic sterol skeleton. Squalene epoxidase is the first enzyme in the pathway that requires molecular oxygen, and this reaction is performed by a microsomal complex consisting of a flavoprotein with NADPH cytochrome C reductase activity, and a terminal oxidase that is not of the cytochrome P-450 family [38]. 

In a reaction that is considered to be one of the most complex in the sterol pathway, 2,2-oxidosqualene cyclase cyclizes the intermediate 2,3-oxidosqualene to lanosterol, the initial precursor of all steroid structures formed by mammals, fungi, and trypanosomatids. 

After the cyclization of 2,3-oxidosqualene to form lanosterol, several sequential transformations occur to form cholesterol in mammals and ergosterol in trypanosomatids and fungi. Some of these reactions are common to all eukaryotes, whereas others are exclusive to each organism, with differences also between trypanosomatids and fungi. 

One of the earliest steps in the lanosterol pathway is the demethylation of the ring system at the C14 position in a two-stage oxidative reaction catalyzed by a cytochrome P-450-containing monooxygenase enzyme known as lanosterol-14*α*-methyl demethylase (C14*α*-demethylase) [38]. 

Removal of the 14*α*-methyl by C14*α*-demethylase generates a Δ^8(14)^ unsaturated sterol with a double bond at the C14 position. This unsaturation needs to be removed to produce Δ^5^ sterols in two consecutive reactions catalyzed by the enzyme Δ^8(14)^-reductase with NADPH as cofactor. In several fungi, this removal is essential, because the subsequent enzymes in the pathway do not metabolize this unnatural sterol. However in some fungal species such as *Candida albicans* [38, 40], and also *L. amazonensis* [11] and *T. cruzi* [42–44], it is metabolized by the C4-demethylase enzymes and Δ^24^-methenylase to form Δ^14^ fecosterol. 

After removal of the C4 and C14 methyl groups and the methenylation of the side chain, the next reaction in the sequence is the isomerization of the double bonds in fecosterol in a reversible reaction catalyzed by the Δ^7^-Δ^8^ isomerase that does not require cofactors such as NADPH.

One of the most important stages of ergosterol biosynthesis that does not exist in the synthesis of cholesterol is the addition of a methyl group at the C24 position in the sterol side chain. Depending on the microorganism, this occurs early after the production of lanosterol, as observed in *L. amazonensis* [11], or at the level of zymosterol (which does not have the C4 and C14 methyl groups in the structure) as found in several fungi [45] and some *Leishmania spp.* [46]. The methyl group is transferred from S-adenosyl-L-methionine (SAM) to C-24 of Δ^24^ sterols to produce Δ^24(28)^-sterols in some reactions catalyzed by the S-Adenosyl-L-methionine:Δ^24^-sterol methyltransferase (EC 2.1.1.43; 24-SMT). 24-SMT is a 150 000 dalton membrane-bound protein that is present in plants, fungi, and trypanosomatids, but is absent in mammalian sterol biosynthetic systems; thus it may constitute an interesting target for the development of antifungal and antitrypanosomal agents. Cell fractionation studies have suggested that 24-SMT is located in the glycosomes and in the mitochondrion [35]. However, immunofluorescence and electron microscopic observations using antibodies generated against the recombinant protein showed that 24-SMT is located in the endoplasmic reticulum and in translucent vesicles that presumably belong to the endocytic pathway [47].

## 3. Available Drugs that Interfere with the Sterol Biosynthetic Pathway

Currently, there are several known drugs that interfere with sterol biosynthesis which are used to treat diseases such as high cholesterol in humans and fungal infections. Table 1 shows the representative compounds distributed in different classes, some of which are commercially available.

Statin is one of the main classes of the sterol biosynthesis inhibitors (SBIs), which act on the mevalonate pathway by the inhibition of HMG-CoA reductase. They have been widely used for cholesterol reduction in humans [38]. A drawback of the statins is their effect on the synthesis of isoprenoid compounds that are essential for several cellular events. Atorvastatin, widely used for treatment of hyperlipidemia, is one example of this class of drugs [48]. 

Bisphosphonate is another important class that interferes with the isoprenoid pathway inhibiting the step catalyzed by farnesyl diphosphate synthase (FPPS). They are used for the treatment of different bone resorption diseases, including osteoporosis, Paget's disease, hypercalcemia caused by malignancy, and tumor metastases in bone [49]. Alendronate and risedronate are two examples of bisphosphonates used for the treatment of osteoporosis and other bone resorption diseases [49]. 

Zaragozic acids and quinuclidines are known inhibitors of squalene synthase (SQS), which catalyzes the first committed step in sterol biosynthesis. This is a very attractive target because its inhibition does not interfere with isoprenoid production and intermediate metabolites that are formed can be readily metabolized and excreted [50]. SQS has been under intense scrutiny with the aim of developing new cholesterol-lowering agents for humans. Previous experimental studies with animals have demonstrated the effectiveness of quinuclidine-based SQS inhibitors such as 3-(biphenyl-4-yl)-3-hydroxyquinuclidine [BPQ-OH] as cholesterol- and triglyceride-lowering agents [51–53]. 

Allylamines are known inhibitors of squalene epoxidase. One good example is terbinafine, which has been shown to be a potent compound against fungi, showing both oral and topical efficacy. Terbinafine inhibits squalene epoxidase leading to a depletion of ergosterol. Importantly, it does not inhibit the mammalian enzyme [38]. 

Azoles are important inhibitors of C14*α*-demethylase, and since they are effective against most fungal diseases, they are presently considered to be the most important antifungal compounds in use. Ketoconazole, one of the first azoles developed, was intensely used for several years. More recently, however, four new commercially available triazoles have been shown to be much more effective: fluconazole, itraconazole, voriconazol, and posaconazole. 

The last class of ergosterol biosynthesis inhibitors comprises the azasterols, which inhibit Δ^24(25)^-sterol methyltransferase (SMT). Inhibition of this step appears to have high selectivity for fungi and trypanosomatids since this enzyme is not found in mammalian cells. Antifungal activities of azasterols have been described for *Candida spp.* [38, 39], *Pneumocystis carinii* [54], and *Paracoccidioides brasiliensis* [55]. 

In conclusion, based on the comments made above it is clear that further exploration of the sterol biosynthesis pathway is highly relevant to the treatment of both chronic diseases, such as hypercholesterolemia, and infectious diseases caused by fungi and trypanosomatids. Therefore, drugs known to inhibit enzymes of the sterol biosynthesis pathway should be tested against all these diseases. Indeed, several drugs developed either to reduce cholesterol levels in humans or to treat fungal diseases have been tested with some success against trypanosomatids, as will be discussed below. We also believe that new drugs shown to be active against trypanosomatids should be tested on fungi as well as in mammals.

## 4. Effects of SB Inhibitors on Trypanosomatid Growth

In *T. cruzi,* inhibitors of HMG-CoA reductase such as mevinolin (lovastatin) have been tested *in vitro* and *in vivo*. Against the extracellular proliferative epimastigote forms, mevinolin produced a dose-dependent reduction of the growth rate up to 25 *μ*M. In the intracellular proliferative amastigote forms only very modest effects were observed up to 0.75 *μ*M, above which a significant effect was also observed in the mammalian host cells. In intracellular amastigotes, it has been shown that supplementation of mevinolin with ketoconazole gives a synergistic effect, so that lower concentrations of azoles become more effective. On the other hand, the combination of mevinolin with terbinafine produced an additive effect, whereas the combination of mevinolin, terbinafine, and ketoconazole showed again a synergistic effect on amastigotes [56]. These results together indicate that a combination of drugs acting in consecutive steps of the sterol biosynthesis pathway may be a promising approach for the treatment of diseases caused by some pathogenic protozoa.

Bisphosphonates have been tested *in vivo* and *in vitro* against different protozoan parasites, including *Leishmania spp.*, *T. cruzi, T. brucei, Plasmodium falciparum*, and *T. gondii* [57–59]. More than 50 new compounds were tested in different protozoan parasites, some of them presenting IC_50_ values lower than 1 *μ*M [60, 61]. In addition, to confirm that the isoprenoid enzymes are involved in the inhibition, they were also tested on the isolated *L. major*, *T. cruzi*, and *T. gondii* farnesyl diphosphate synthase enzymes (LmFPPS, TcFPPS, and TgFPPS). A potent inhibition of the enzyme's activities indicated that some of them are specific for inhibition of the isoprenoid pathway, thus validating this pathway as an interesting and selective drug target for these parasites. Garzoni et al. [62] reported that risedronate showed a selective effect against *Trypanosoma cruzi*, leading to complete growth arrest and lysis at 150 *μ*M for epimastigotes. Complete destruction of intracellular amastigotes was observed at 100 *μ*M risedronate, thus preventing the development of *T. cruzi* infections in murine muscle heart or in Vero cells [62].

**Figure 4:**
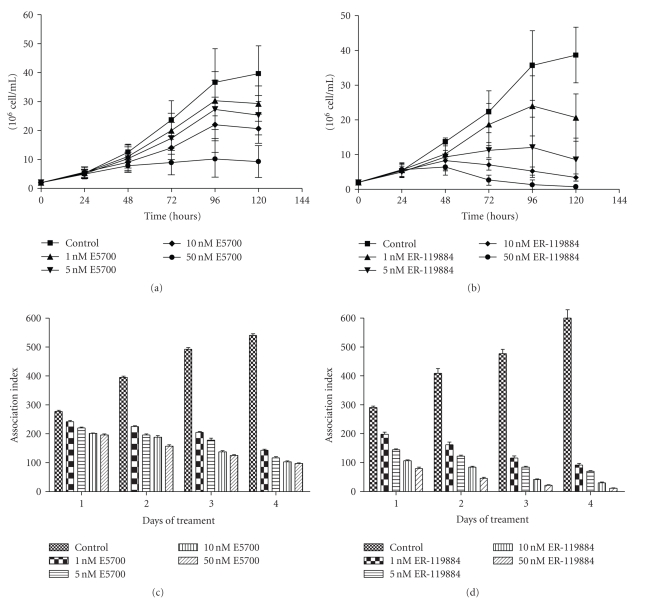
(a)-(b) Growth curves of promastigotes and (c)-(d) intracellular amastigotes of *Leishmania amazonensis* treated with two potent squalene synthase inhibitors, E5700 and ER-119884. The graphics are reproduced with permission from [37] American Society for Microbiology.

In trypanosomatids and fungi, there are several works describing the potent and selective activity of zaragozic acids and quinuclidines [35–37, 63–68]. For example, ER-119884 and E5700, two novel quinuclidine derivatives produced by Eisai Co. (Tokyo, Japan), have been shown to be potent anti-*Trypanosoma* and *Leishmania* agents *in vitro*, leading to a dramatic depletion of the parasite's endogenous sterols, that is, associated with an intense antiproliferative activity [36, 37]. Figures 4(a)–4(d) shows the antiproliferative effect of E5700 (Figures 4(a)  and 4(c)) and ER-119884 (Figures 4(b)  and 4(d)) in promastigotes and intracellular amastigotes of *Leishmania amazonensis*. These compounds are very potent against both forms of the life cycle, presenting MIC values of 30 and 10 nM for promastigote, and 2.0 and 0.5 nM for intracellular amastigotes [37]. When compared with the minimal concentration that affects macrophages, these values are, respectively, 100 000 and 25 000 fold greater than the corresponding IC_50_, indicating that they are selective against the parasite without any effect in the host cells. 

In *Leishmania*, Vannier-Santos et al. [70] showed that terbinafine is able to interfere with the growth of promastigotes and intracellular amastigotes, inducing dramatic changes in their structural organization, especially in the mitochondrion. For terbinafine, the IC_50_ values were around 1 *μ*M for promastigotes and 100 nM for intracellular amastigotes. However when in combination with ketoconazole, which also interferes with ergosterol biosynthesis, the values decreased to around 1 nM for intracellular amastigotes, indicating once more that the approach of inhibiting multiple steps of this pathway is a promising alternative for chemotherapy [70].

In trypanosomatids, azoles have been used *in vitro* against *T. cruzi* and *Leishmania*. Drugs such as D0870 and posaconazol were tested in vitro and *in vivo* with positive and interesting results, and also have a potent effect against acute and chronic experimental Chagas' disease [69, 71–75]. Ketoconazole was also tested alone or in combination with other sterol biosynthesis inhibitors [42, 44, 71, 76]. The IC_50_ of Posaconazole for *T. cruzi* epimastigotes and amastigotes was 3 and 0.25 nM, respectively. However when in combination with amiodarone, a K^+^ and Ca^2+^ channel antagonist, they presented synergistic effects with a fractional inhibitory concentration (FIC) of 0.42 *μ*M [73]. Amiodarone was also tested in *Leishmania mexicana*, having an IC_50_ similar to that found for *T. cruzi*, due to alterations in the physiology of the mitochondrion and acidocalcisomes [77]. 

The effects of azasterols on *T. cruzi* [4, 42–44, 78], *Leishmania* [5, 11, 47, 78, 79], and *T. brucei* [16] have been extensively studied. The antifungal activities of azasterols against *Candida spp.*, *P. carinii* [54], and *P. brasiliensis* [55] have also been described. We have shown that several azasterols are active against *L. amazonesis* with IC_50_s in the submicromolar to nanomolar range, indicating that this step has potential as a chemotherapeutic target [11, 78, 80]. Furthermore, when in combination with azoles they are even more effective, and sometimes acting synergistically [43].

## 5. Effects of SB Inhibitors on the Ultrastructure of Trypanosomatids

It has been shown that ergosterol biosynthesis inhibitors induce dramatic alterations in the ultrastructure of several organelles [11, 37, 64, 66, 70, 74–76, 78–81]. These alterations occur mainly in the mitochondrion-kinetoplast complex, the endoplasmic reticulum, the Golgi complex, nucleus, multivesicular structures, lipidic inclusions, the contractile vacuole, and also in the plasma membrane covering the cell body, and the flagellum.

**Figure 5:**
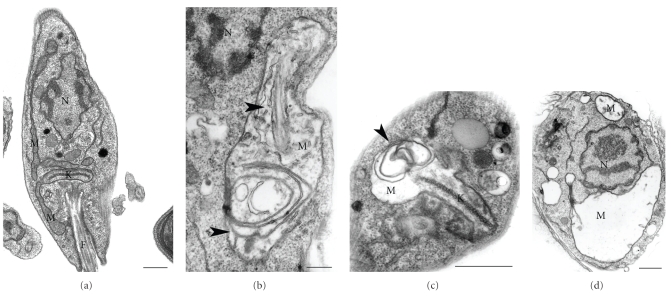
Ultrathin sections of *L. amazonensis* promastigotes control (a) and treated with azasterols, known inhibitors of the Δ^24(25)^-sterol methyltransferase (b)–(d). (a) General overview of an untreated-parasite showing a normal ultrastructure of the mitochondrion (M), kinetoplast (k), flagellum (F) and nucleus (N). (b)–(d) Treated-parasites presenting severe alterations in the mitochondrion structure such as a disorganization of the internal membranes ((b) and (c), *arrowheads*) and an intense and evident mitochondrial swelling with loss of the matrix content (b)–(d). Bars 0.5 *μ*m.

**Figure 6:**
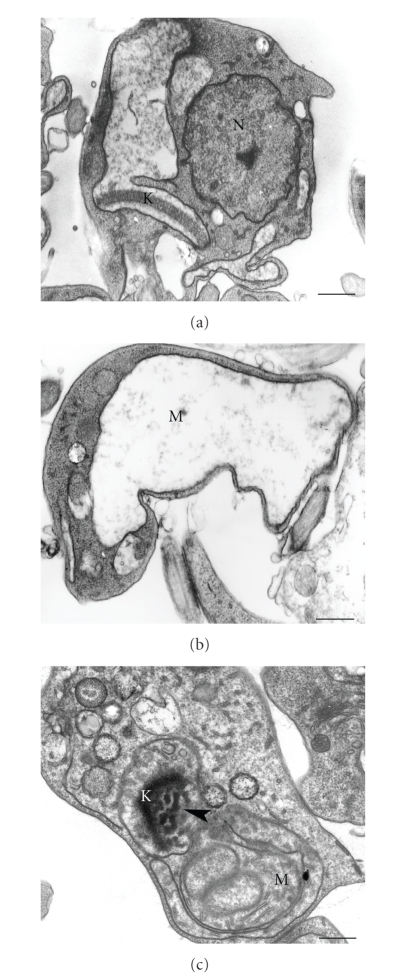
*Trypanosoma cruzi* epimastigotes treated with ketoconazole, an inhibitor of the C14*α*-demetilase (a)-(b), and *L. amazonensis* promastigotes treated with E5700, an inhibitor of the squalene synthase (c) showing an intense mitochondrial swelling (a)-(b) and alterations in the kinetoplast structure ((c), *arrowhead*). K, kinetoplast; M, mitochondrion. (a)-(b) Images are reproduced with permission from [74] American Society for Microbiology. Bars, 0.5 *μ*m.

The mitochondrion-kinetoplast complex has been shown to constitute an important organelle target of drugs that inhibit sterol biosynthesis. It is important to point out that *T. cruzi* and *Leishmania* have only one highly ramified mitochondrion distributed throughout the protozoan body (Figure 5(a)) (reviewed in [82]). Treatment of *L. amazonensis* with different azasterols induced mitochondrion alterations such as a disorganization of the mitochondrial membranes (Figures 5(b)-5(c)) followed by an intense swelling and loss of the matrix contents (Figures 5(b)-5(d)) [11, 76]. These alterations in the mitochondrion were also observed after treatment of *T. cruzi* and *L. amazonensis* with terbinafine, ketoconazole, and ICI195,739 [70, 74–76]. The swelling is dramatic and the mitochondrion appears to occupy the whole cytosol in epimastigotes treated with ketoconazole (Figures 6(a)-6(b)). The mitochondrial alterations were confirmed by measuring the mitochondrial membrane potential in digitonin-permeabilized parasites [79]. On the other hand, the inhibitors did not affect the macrophage's mitochondria, which can be visualized using JC-1, a cationic mitochondrial vital dye [37]. Most probably all these morphological changes are due to modifications in the composition of the mitochondrial membranes due to interference with the synthesis of ergosterol and accumulation of intermediate metabolites [83]. Lipid analysis performed in epimastigotes of *T. cruzi* showed that the mitochondrion has a different lipid composition when compared with mammalian cells, including the presence of ergosterol, thus explaining the potent effect of SB inhibitors on its ultrastructure and physiology [84]. 

One characteristic feature of the unique mitochondrion of trypanosomatids is the presence of a complex network of DNA localized in a portion of the mitochondrion. This network is located just below the basal bodies from which the flagellum is formed, and is known as the kinetoplast (reviewed in [85]). The kinetoplast DNA is organized as thousands of concatenated minicircles and a few maxi-circles. It was shown that after treatment with E5700, an SQS inhibitor, alterations were observed in the kinetoplast structure of *L. amazonensis*. In treated cells, the kinetoplast appeared completely disorganized relative to its normal structure (Figure 6(c)). These alterations probably result from changes that take place in the organization of the inner mitochondrial membrane, that is, connected to the kinetoplast DNA network.

**Figure 7:**
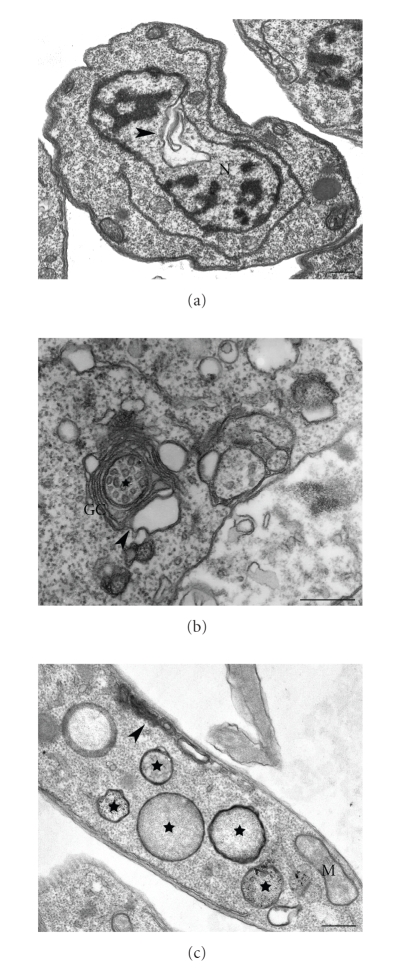
*L. amazonensis* promastigotes treated with azasterols (a)-(b), and ER-119884 (c) showing alterations in the nuclear membrane ((a), *arrowhead*), in the Golgi complex ((b), *arrowhead*), and in the endoplasmic reticulum ((c), *arrowhead*). In the Figures 6(b) and 6(c), the presence of a multivesicular bodies and autophagosome-like structures (*stars*) could be related with a remodeling process of damaged organelle by autophagy. GC, Golgi complex; M, mitochondrion; N, nucleus. Bars, 0.5 *μ*m.

**Figure 8:**
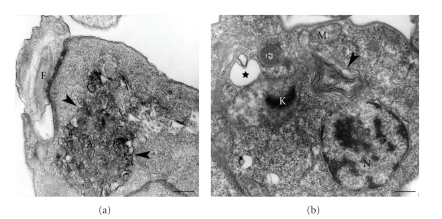
Promastigotes treated with quinuclidine and azasterol, respectively, showing the presence of structures related with autophagy such as a large vacuole containing many membrane profiles ((a), *arrowheads*), and a myelin-like figures involving part of the cytosol ((b), *arrowhead*). Star indicates the presence of a possible contractile vacuole near the flagellar pocket. A, autophagosome; F, flagellum; K, kinetoplast; M, mitochondrion; N, nucleus. (a) This image is reproduced with permission from [66] Elsevier. Bars, 0.25 *μ*m.

Alterations in the nuclear membrane, Golgi complex and endoplasmic reticulum were also observed after treatment with different SB inhibitors (Figures 7(a)–7(c)). The presence of a multivesicular body associated with the trans-Golgi network can be seen in Figure 7(b) (star), thus suggesting alterations in the secretory pathway. Furthermore, Figure 7(c) (stars) also shows the presence of some vacuoles resembling autophagosomes, thus suggesting the occurrence of cell death by autophagy (reviewed in [86–88]). The presence of large vacuoles containing membrane profiles, endoplasmic reticulum forming myelin-like figures or engulfing a part of the cytoplasm, and damaged organelles supports the idea that an autophagy-like process takes place in these cells (Figures 8(a)-8(b)).

**Figure 9:**
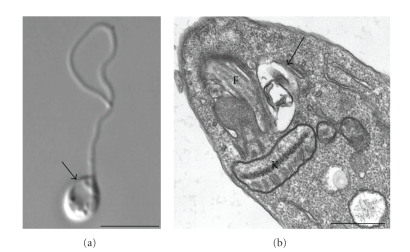
(a) Diferential interference contrast (DIC) microscopy and (b) transmission electron microscopy showing the presence of a prominent contractile vacuole (arrows) near the flagellar pocket after treatment of *L. amazonensis* promastigotes with quinuclidine inhibitors. In the left panel it is possible to observe a rounded and swollen parasite that probably indicates osmotic changes due alterations in the plasma membrane's permeability. F, flagellum; K, kinetoplast. Bars, 5 *μ*m and 0.5 *μ*m, respectively.

Recent studies using various microscopy techniques have shown that trypanosomatids possess a structure located close to the flagellar pocket identified as a contractile vacuole (reviewed in [89]). This structure became much more evident in *L. amazonensis* (Figures 9(a)-9(b)) and *T. cruzi* epimastigotes [64] treated with SB inhibitors. As observed by differential interference contrast microscopy, the treated cells appeared rounded and swollen (Figure 9(a)), suggesting osmotic changes, thus explaining the presence of a prominent contractile vacuole. These changes may be due to alterations in the plasma membrane's permeability to ions induced by the complete depletion of sterols and sterol-like molecules, which is likely to lead to significant changes of the physicochemical properties of the lipid bilayer [90]. 

**Figure 10:**
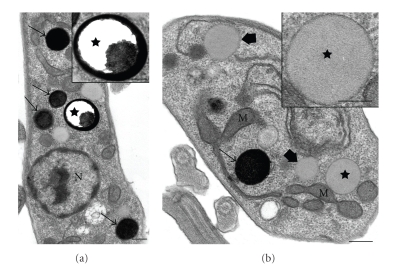
Treatment of promastigotes with ER-119884 induces the accumulation of several lipid droplets in the cytosol, sometimes appearing as an electrondense structure due to osmium tetroxide concentration ((a)-(b), *small arrows*), and as a classic lipid body surrounded by a phospholipid monolayer ((b), *large arrows*). At high magnification (*stars*), it is evident that the structures are completely different, probably indicating a distintic nature of the lipids that accumulate in these inclusions. M, mitochondrion; N, nucleus. All images are reproduced with permission from [37] American Society for Microbiology. Bars, 0.5 *μ*m.

Another important alteration observed after treatment with SB inhibitors is the presence of several lipid droplets displaying variable morphology, as shown in Figures 10(a)-10(b). The formation of these structures is probably due to accumulation of lipid precursors. The images showed a large variation in the electron density of the structures, suggesting that different types of lipids are accumulated. Some appeared very dense after postfixation with osmium tetroxide (Figure 10(a)). Others, however, appeared as electron-lucent structures (Figure 10(b), large arrows and stars). They are surrounded by a typical monolayer of phospholipids (high magnification in Figure 10(b)). These lipid droplets could be also identified by fluorescence microscopy using the neutral lipid marker Nile Red [37]. Lipid droplets were also observed in *L. major* null mutants for important enzymes of the sphingolipid biosynthesis pathway [91, 92], and in *T. cruzi* after perturbation of the sphingolipid content [93]. On the other hand, they also accumulated when epimastigotes were treated with different classes of inhibitors, including cytoskeletal inhibitors [94], indicating that lipid body formation can occur as a consequence of perturbations in parasitic functions not related to lipid biosynthesis. 

**Figure 11:**
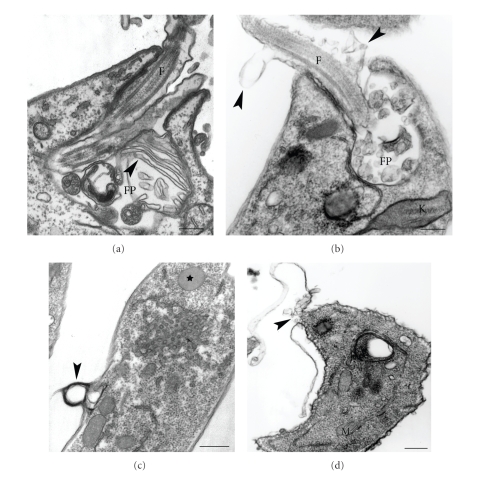
Ultrathin sections of *L. amazonensis* promastigotes treated with different sterol biosynthesis inhibitors showing severe alterations in the plasma membrane lining (a) the flagellar pocket, (b) the flagellum, and (c)-(d) the cell body. In (d) it is possible to observe a breakdown in the plasma membrane and release of the subpellicular microtubules (*arrowhead*). Star in (c) shows a classic lipid body. F, flagellum; FP, flagellar pocket; k, kinetoplast; M, mitochondrion. Images are reproduced with permission from [79] (a), and [66] (b), (d) Elsevier. Bars, 0.25 *μ*m (a)–(c) and 0.5 *μ*m (d).

Alterations in the plasma membrane lining the cell body, flagellar pocket and flagellum were also observed, but the morphological changes varied according to the inhibitor (Figures 11(a)-11(b)). When the parasites where incubated with azasterols, alterations in the flagellar pocket were predominant (Figure 11(a)), while SQS inhibitors induced alterations mainly in the membrane lining the cell body (Figure 11(c)-11(d)) and sometimes in the flagellar membrane (Figure 11(b)) [64]. These different phenotypes suggest that the three domains of the plasma membrane have distinct lipid compositions. On the other hand, the presence of membrane blebs could be related to apoptosis-like death [86].

**Figure 12:**
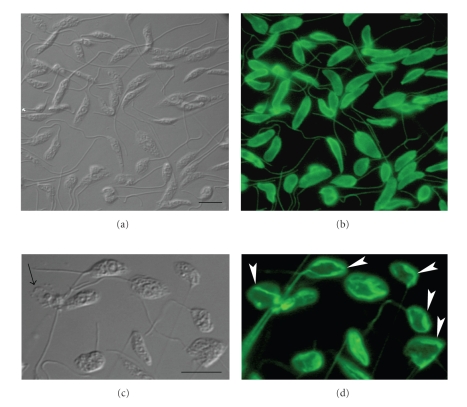
DIC microscopy (left panel) and immunofluorescence microcospy (right panel) of *L. amazonensis* promastigotes control (a)-(b) and treated with ER-119884 (c)-(d). The labeling corresponds to the cytoskeleton constituted mainly by tubulin, revelead here by using of an Alexa 488-labeled secondary antibody. The black arrow in DIC image corresponds to the cell body which sometimes appeared changed and rounded as compared to the control parasites, while the white arrows point to several tubulin clusters that accumulated in the cytosol after treatment. All images are reproduced with permission from [37] American Society for Microbiology. Bars, 5 *μ*m.

It is well known that in trypanosomatids there is a close connection between the plasma membrane lining the cell body and the subpellicular microtubules, and that the spatial distribution of these microtubules is responsible for the maintenance of the protozoan's shape (reviewed in [95]). Immunofluorescence microscopy of tubulin-stained trypanosomes treated with an SBI revealed changes in the shape of the cell and in the distribution of the subpellicular microtubules, probably due to alterations in the sterol composition of the plasma membrane [37]. Figures 12(a)–12(d) show the effect of ER-119884, an SQS inhibitor, on the cytoskeleton of *L. amazonensis* promastigotes. The changes were also seen by transmission electron microscopy (Figure 11(d)). 

**Figure 13:**
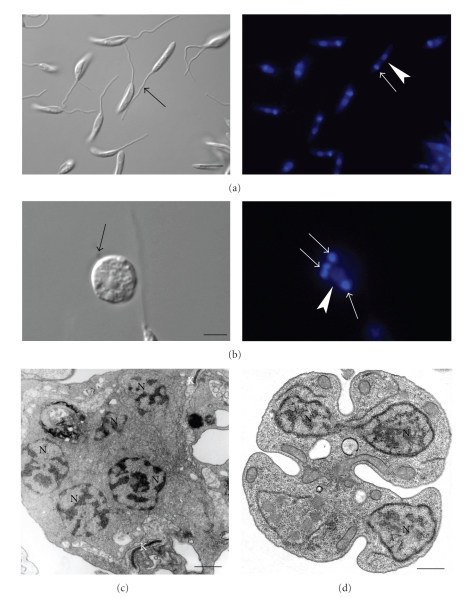
DIC microscopy ((a)-(b), *left panel*), fluorescence with DAPI ((a)-(b), *right panel*) and transmission electron microscopy (c)-(d) of treated-promastigotes to evidenciate the alterations in the cell cycle after treatment with BPQ-OH and ER-119884. (a) Control cells present a correct number of kinetoplast (white arrow), nucleus (arrowhead), and flagellum (black arrow), one of each for a unique cell. (b) After treatment, the number of these organelles is completely alterated and it is possible to find cells with one flagellum (black arrow), four kinetoplast (white arrows), and one nucleus (arrowhead). (c)-(d) Alterations in the cell cycle can also be evidenced by transmission electron microscopy with the appearance of cells with several nuclei and kinetoplasts. K, kinetoplast; N, nucleus. These images are reproduced with permission from [37] American Society for Microbiology (a), (b), (d), and from [66] Elsevier (c). Bars, 5 *μ*m (a)-(b), and 0.5 *μ*m (c)-(d).

Recent observations show that SBIs also interfere with the protozoan cell cycle. Using fluorescence microscopy of cells stained with DAPI to label the nucleus and kinetoplast, as well as transmission electron microscopy, it was shown that ER-119884 and BPQ-OH interfere dramatically with the cell cycle, inducing several abnormal phenotypes, including cells with multiple nuclei, kinetoplasts, and flagella (Figures 13(a)–13(d)). The effect on the cell cycle was already evident after 24 hours of incubation in the presence of the inhibitors, and the number of cells containing abnormal numbers of flagella, kinetoplasts, and nuclei increased with time [37]. There are at least two possible explanations for these effects: (i) the cells do not complete cell division due to the depletion of essential endogenous sterols, which control the dynamics of the membrane but are also key regulators of the cell cycle [96, 97], or (ii) the organization of the cytoskeleton necessary for the completion of cytokinesis, which requires interactions with the nuclear membrane, is in some way affected by SQS inhibitors. In addition, SBI also induced significant alterations in the trypanosomatids' nuclei. 

**Figure 14:**
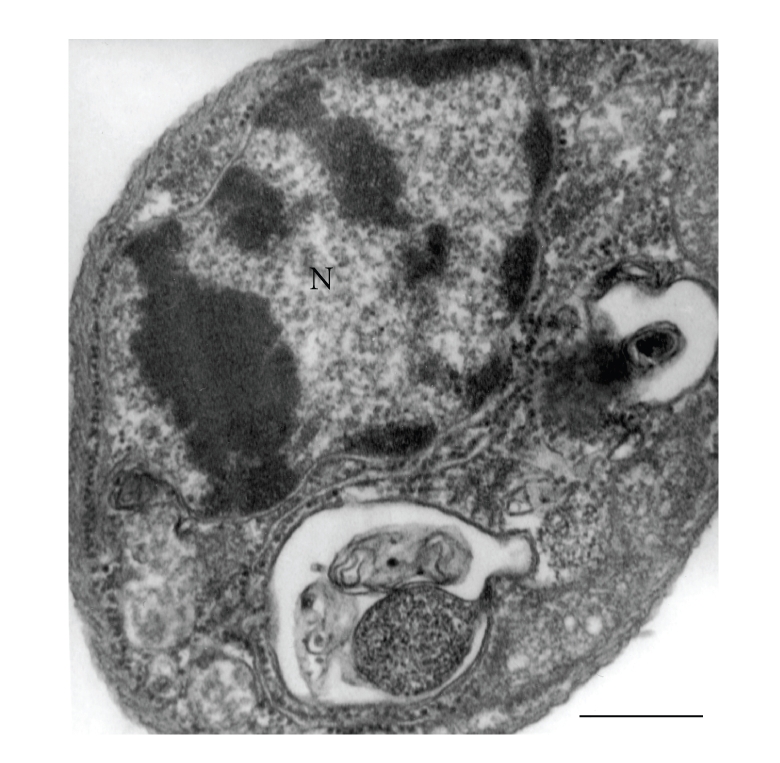
*Leishmania amazonensis* promastigote treated with sterol biosynthesis inhibitors showing a condensation of the nuclear chromatin, a characteristic feature of the apoptosis-like cell death process. N, nucleus. Bar, 0.5 *μ*m.

A significant number of treated cells showed abnormal chromatin condensation (Figure 14), indicating a process of cell death by apoptosis (reviewed in [86–88]).

All these alterations discussed here indicate that sterols play an essential role in a significant number of cellular processes, including the cell cycle, cell death, and the maintenance of the membrane's structure, stability, and function.

## 6. Effects of SB Inhibitors on Lipid Composition

It is now well established that SB inhibitors lead to the accumulation of intermediates of the ergosterol biosynthesis pathway (reviewed in [4]). Treatment of *T. cruzi* epimastigotes with mevinolin, an inhibitor of HMG-CoA reductase, led to a significant reduction in the presence of ergosterol-like molecules, with a concomitant increase in exogenous cholesterol [56].

Incubation of the parasites with bisphosphonates, inhibitors of the farnesyl diphosphate synthase (FPPS), induces an accumulation of isopentenyl diphosphate (IPP) and inhibits the formation of farnesyl diphosphate (FPP). Consequences of this inhibition include a decrease in the level of protein prenylation, and reduced production of molecules such as dolichols, ubiquinones, heme a, and sterols (reviewed in [58]).

The inhibition of squalene synthase (SQS) induces a total depletion of squalene, endogenous ergosterol, and other 24-methyl sterols, and these are completely replaced by exogenous cholesterol [35–37]. This potent effect in the sterol composition is consistent with the inhibition of SQS enzyme in concentrations near the nanomolar to subnanomolar range, in a reaction that is noncompetitive with the substrate [37]. 

Treatment of *L. mexicana* promastigotes with terbinafine, a potent inhibitor of squalene-2,3-epoxidase, results in the accumulation of squalene, and a reduction in the amount of endogenous C_28_- and C_29_-sterols [98]. In *T. cruzi* epimastigotes the same treatment led to the depletion of sterols and accumulation of phosphorylated hydrocarbons, with a lower amount of squalene when compared with *Leishmania* [99]. The K^+^ and Ca^+2 ^ channel antagonist amiodarone also induces the inhibition of squalene epoxidase in *T. cruzi* and *L. mexicana*, leading to an accumulation of squalene and complete depletion of 24-methyl sterols such as ergosterol and 5-dehydroepisterol [73, 77].

Incubation of the parasites in the presence of azoles, which inhibit C14*α*-demethylase, led to the replacement of normal endogenous sterols by various 14*α*-methyl sterols. In this situation, the first sterol that accumulates in promastigotes of *Leishmania* is 4*α*,14*α*-dimethyl-dimethylzymosterol; however, after a long exposure this sterol molecule could be metabolized by 24-SMT, producing sterols alkylated at C-24, or demethylated at C-4 by C4*α*-demethylase to produce 4-desmethylsterol [11, 46, 100]. In *T. cruzi* epimastigotes and amastigotes, the accumulating 14*α*-methylsterol is lanosterol, particularly its C-24 alkylation product (24-methylenedihydrolanosterol) [5, 14, 69, 71–75, 99, 101]. Unlike *Leishmania*, demethylation at C4 seems to be very restricted in *T. cruzi,* and the C4*α*-demethylase has higher specificity for sterols after the removal of the 14*α*-methyl. On the other hand, treatment with SCH 56592 (posaconazole) also causes an accumulation of squalene, possibly indicating that some regulatory step involved in squalene cyclization is regulated by high amounts of lanosterol and its 24-methylene derivatives [72]. 

The C-24 transmethylation reaction catalyzed by 24-SMT in trypanosomatids is inhibited by various azasterols that have a nitrogen substitution in the side chain. This leads to a depletion of C_28_-sterols such as ergosterol, episterol, and 5-dehydroepisterol, which are then replaced by large amounts of zimosterol and cholesterol ingested from the culture media [11, 42, 43, 102] through the endocytic pathway [3]. Furthermore, a simultaneous incubation with azasterol and ketoconazole induces an accumulation of lanosterol, 4,14-dimethyl-zymosterol, and 14-methyl-zymosterol [11, 42, 43], indicating that the mechanism of action of 24-SMT in trypanosomatids is similar to that observed in fungi, yeasts, and plants [32, 38, 44, 103–105]. These results also indicate that in some trypanosomatids, 24-SMT can use zymosterol or its 14-methyl or 4,14-dimethyl derivatives as substrates. In the case of fungi, the substrate for 24-SMT is restricted to zymosterol [32, 38]. On the other hand, experiments exposing *Leishmania spp.* to low concentration azasterol for a long time showed that they are able to survive by modulating biosynthesis to use C_27_-sterols as substrates for 24-SMT [102]. Thus, these biochemical analyses indicate that in the case of sterol biosynthesis it is better to use combination therapy to inhibit more than one step in order to completely eliminate all the sterol substrates, particularly of the 24-SMT enzyme, which participates in essential reactions. 

It is important to point out that in all biochemical analyses of the lipid composition of trypanosomatids after drug treatment, a significant accumulation of cholesterol is observed in both *T. cruzi* and *Leishmania spp.,* indicating that the parasites try to compensate for the absence of endogenous sterols. It is also clear that 24-methyl and/or 24-ethyl sterols are essential for the maintenance of membrane structure and function, as well as other vital cellular process like the cell cycle.

## 7. Effects of SB Inhibitors in Experimentally Infected Animals and Perspectives for Human Therapy

Several of the SB inhibitors have been tested using murine models of Chagas' disease, leishmaniasis, and malaria. Treatment of acute Chagas' disease with mevinolin (lovastatin) in combination with ketoconazole led to the elimination of circulating parasites and complete protection against death in the murine model [56]. 

Bisphosphonates were also tested in acute Chagas' disease as well as in leishmaniasis experimental models. In *Leishmania* infections, recent studies showed that pamidronate is able to promote a radical cure of experimental cutaneous leishmaniasis in mice [105], and also that it is active *in vivo* against *L. donovani* by intravenous administration [106]. Furthermore, *in vivo* studies using the murine model of acute Chagas' disease showed that treatment with 1 mg/kg risedronate per day for 7 days induced a more than 90% reduction in parasitemia and significantly increased the animals' survival. On the other hand, at higher concentrations (up to 10 mg/kg per day), risedronate led to a reduction of parasitemia and mortality with no toxic effects for the treated animals [107]. 

Inhibitors of squalene synthase such as ER-119884 and E5700 were also tested in a murine model of Chagas' disease, revealing that E5700 is able to provide full protection against death and to completely suppress parasitemia, with no toxicity to the host [36]. 

Some azoles were also tested against *T. cruzi* and *Leishmania spp.* with interesting results. D0870, a bis-triazole derivative, showed a potent effect, preventing death and inducing parasitological cure in 70 to 90% of animals in acute and chronic murine models of Chagas' disease [71, 108]. SCH 56592 (posaconazole), which is one of the new triazole derivatives, has been tested against Chagas' disease [72, 109] and leishmaniasis [110]. In the acute Chagas' disease model, 43 doses of ≥10 mg/kg of body weight/day promoted 85 to 100% survival, with 90 to 100% cure of the animals as verified by parasitological, serological and PCR-diagnostics. By contrast, ketoconazole at 30 mg/kg/day gave only a 60% survival rate and a 20% cure rate. [72]. In the chronic phase, the results were also positive, with 85% of the animals protected from death and 75% parasitologically cured. In addition, the combination of posaconazole with amiodarone produced a delay in the development of parasitemia after a high rate of infection mice, and in mice with a lower infection rate, the survival and cure were higher when compared with posaconazole alone [73]. Amiodarone alone also led to a decrease in parasitemia and a 40% increase in the survival of the infected animals [73]. On the other hand, in cutaneous leishmaniasis, treatment with 60 and 30 mg/kg/day, of SCH 56592 was highly efficacious, and the higher dose was superior to amphotericin B at a dose of 1 mg/kg/day [110]. In visceral leishmaniasis due to *L. donovani* infection, treated mice showed a significant reduction in parasite burden in the liver and spleen compared to untreated mice [110]. 

*In vivo* studies with 22,26-azasterol, one of the most potent inhibitors of 24-SMT, have shown that it has selective antiparasitic activity in a murine model of acute Chagas' disease [43].

## 8. Perspectives

It is clear that the sterol biosynthesis pathway plays a key role in the metabolism of eukaryotic cells. The observation that compounds found in several steps of this metabolic pathway plays essential roles in several basic physiological processes spurred several groups to further analyze this metabolic pathway. From the parasitological point of view, it will be important to test new drugs developed for other medical purposes, such as control of blood cholesterol levels in humans, against trypanosomatids. The same assumption is valid for drugs interfering with the SB pathway in fungal cells. On the other hand, since trypanosomatids, fungi and mammals share several steps of the SB pathway, the trypanosomatids may constitute a useful biological system for the screening of candidate SB inhibitors, and thereby facilitate the search for cholesterol-control drugs for humans. Finally, we expect that new studies will appear providing a better characterization of the last steps of the SB pathway, especially those involved in transformation of zimosterol into ergosterol.

## Figures and Tables

**Table 1:** Representative compounds that interfere with sterol synthesis in eukaryotes, fungi and trypanosomatids.